# Co-Developing Content Updates for the Card Sort Task for Self-Harm–Digital (CaTS-D) With People With Lived Self-Harm Experiences: Pilot Study and Thematic Analysis

**DOI:** 10.2196/71296

**Published:** 2025-10-24

**Authors:** Katherine Bird, Ian Greentree, Brian O'Shea, Ellen Townsend

**Affiliations:** 1 School of Psychology, University of Nottingham, East Drive, Nottingham, NG7 2RD, United Kingdom, 44 07983825563; 2 Greentree Discoveries LTD, Corby, United Kingdom; 3 The Centre for the Experimental-Philosophical Study of the Discrimination, Aarhus University, Aarhus, Denmark

**Keywords:** CaTS, card sort task for self-harm, self-harm research tool, self-harm, self-harm prevention, qualitative, pilot and feasibility testing, online self-harm research tools

## Abstract

**Background:**

Self-harm is a significant global concern with multiple negative outcomes. Self-harm research tools typically focus on single risk factors, meaning the temporal interplay between factors and their impact on self-harm is unknown. The Card Sort Task for Self-Harm (CaTS) addressed these deficits by using 117 cards to examine multiple self-harm factors. In-person research is time-consuming, costly, and limits participation opportunities. Developing an electronic version of CaTS (Card Sort Task for Self-harm—digital; CaTS-D) is necessary to address these issues, capture large datasets, and provide a stronger evidence base. Since CaTS’ inception, understanding of self-harm has evolved, including increasing awareness that lesbian, gay, bisexual, transgender, queer, intersex, asexual, and other minoritized gender and sexual identities (LGBTQIA+) people are at high risk. Updating CaTS is essential to ensure its relevance to both LGBTQIA+ and cisgender-heterosexual self-harm.

**Objective:**

We aimed to present results from two studies. Study 1 is a pilot study assessing the feasibility of CaTS-D. Study 2 used qualitative interviews to identify additions or amendments to CaTS to increase its relevance as understanding of self-harm evolves.

**Methods:**

Study 1 recruited UK residents (N=13, aged 18‐30 y) with lived self-harm experience. Feasibility and acceptability of CaTS-D were assessed using the Systems Usability Scale (SUS) and visual analog scale (VAS). Study 2 recruited UK residents (N=13; LGBTQIA+ n=9 and cisgender-heterosexual n=4; aged 21‐29 y) with lived self-harm experience to one-on-one interviews.

**Results:**

Study 1 found CaTS-D to be a feasible web application for use in self-harm research. VAS data showed no significant difference between pre- and poststudy mood (*t*_9_=1.59; *P*=.15). In Study 2, thematic analysis resulted in 13 additional cards (eg, “Before 6 mo;” “I don’t feel comfortable in my body;” “I was bullied on social media;” and “Self-harm gave me a feeling of control”). Cards were worded clearly, but minor amendments to wording on cards to increase LGBTQIA+inclusivity were identified (ie, changing “boyfriend/girlfriend” to “partner”). While participants felt selective additions were necessary, too many may overwhelm participants. Therefore, future additions should be carefully considered.

**Conclusions:**

Pilot-testing shows CaTS-D is a usable, feasible web application to examine self-harm and capture large datasets. Importantly, completing CaTS-D does not negatively impact participants’ mood. Updates and key additions were made to CaTS from consultation with people with lived self-harm experiences. These increase the relevance of CaTS and ensure LGBTQIA+ inclusivity.

## Introduction

### Background

Self-harm (here defined as any self-injury or poisoning regardless of intent) [1,2] is a global public health concern [3] and the result of complex, temporal interactions between various biological, social, and psychological factors [4]. Self-harm is a multifaceted [5] and prevalent [6] behavior linked to several maladaptive health, social, and well-being outcomes. For example, drug and alcohol misuse [7,8], repeated self-harm [8,9], and reduced education and employment outcomes [10]. Most concerningly, self-harm is also a key risk factor for future suicidal thoughts and behaviors [11] and the most significant risk factor for suicide [12,13]. A recent large-scale study found suicide risk was 49 times greater in the year following self-harm [4]. Globally, suicide is a leading cause of death [14]. In 2019, approximately 703,000 people died by suicide [15]. Considering self-harm is a key antecedent for suicide, reducing self-harm is an important suicide prevention strategy and is included in the National Suicide Prevention Strategy for England and Wales [16,17]. People experience a multitude of risk (ie, hopelessness [11] and alcohol use [5]) and protective (ie, social and family support [18] and self-compassion [19]) factors for self-harm. However, historically, factors have been investigated in isolation [5,20]. This limits our understanding of the temporal dynamics between multiple self-harm factors and whether co-occurring factors increase or mitigate self-harm risk. Understanding complex temporal dynamics between risk and protective factors is recommended by researchers [20-22]. The Card Sort Task for Self-Harm (CaTS) was developed to address these issues.

### The CaTS

CaTS examines multiple factors for self-harm using a set of cards with self-harm–related concepts. Participants use relevant cards to describe their self-harm pathway over time using 7 timepoint cards (6 months before, 1 month before, 1 week before, 1 day d before, 1 hour before, I self-harmed, and afterwards) and 106 cards with self-harm–related thoughts, behaviors, feelings, events, and self-harm supports and services. Concepts used in CaTS were developed from self-harm literature, theory, and models of self-harm, and with academics and clinicians in the field (see [5] for details on the development of CaTS). Example concepts include “I was drinking alcohol,” “I was angry,” “I felt better after self-harm,” “I was bullied,” “I talked to a friend which helped,” and “I trusted a caregiver.” Blank cards enable participants to enter personally relevant concepts not included. See Section S1 in Multimedia Appendix 1 for the complete list of CaTS cards. Concepts were placed into broad groups (ie, feelings and events) to reduce participants being overwhelmed by many cards rather than being representative of superordinate factors. Participants complete CaTS during in-person research [5,23-25] by placing cards relevant to their self-harm under relevant timepoints, usually for their most recent or first ever self-harm episode. Multiple card options enable CaTS to capture the complex pathway to self-harm and demonstrate the pathway is sequential in nature [5,25]. Previously, researchers have used CaTS to examine self-harm pathways in adolescents [5], young people [24], and children in public care [25], and to compare self-harm antecedents in young adults compared to older adults [26].

### CaTS: Developing a Digital Research Tool

CaTS has some limitations. First, in-person research is time-consuming for both researchers and participants and limits recruitment opportunities. To address this, we developed a web application version of CaTS, called CaTS-Digital (CaTS-D). Digital tools provide a flexible, cost-effective option for researchers using technologies most individuals use daily [27]. They are also a useful, effective way to reach large and representative samples [28]. Indeed, approximately 95% of people report owning a smartphone [29,30] and 45% of young people report being online almost constantly [29]. Furthermore, many people are engaged and comfortable [31] using smartphone “apps,” suggesting research undertaken online is likely a favorable option. Digital tools also benefit researchers. For example, web applications can reduce response burden [32,33], which may result in more valid responses [28]. There is also evidence online research increases participant diversity [34]. Furthermore, online methods may reduce recruitment and research costs while increasing recruitment efficiency [28]. Therefore, CaTS-D may be an efficient, versatile, accessible self-harm research tool allowing researchers to capture large, diverse datasets. CaTS-D will also increase the efficiency of data management and analysis procedures.

Second, a digital prototype of CaTS was created following patient and public involvement that identified participants would favor a digital version [26]. Lockwood and colleagues [26] reported similar sequential results to in-person CaTS research suggesting CaTS-D is capable of capturing data comparable to the in-person version. Finally, the existing CaTS version only allows participants to use each card once. During previous in-person research (ie, [5]) participants were asked to place cards under the most pertinent timepoint. However, items may be relevant at multiple or all timepoints. Consequently, research using CaTS may not provide a complete picture of self-harm antecedents, or which factors occur more frequently than others. CaTS-D would address this deficit by allowing participants to select multiple time points for each card and better document their self-harm pathway. This would improve our understanding of self-harm and its antecedents and provide researchers with a stronger data set on which to base recommendations.

### CaTS: Amendments, Updates, and Additions

Since the creation of CaTS in 2013, novel factors have emerged, which need including to ensure CaTS remains a relevant and appropriate research tool. For example, recent systematic [35] and narrative [36] reviews reported emerging evidence of both positive and negative effects of social media usage on self-harm. In addition, evidence indicates self-isolation, financial hardship, and loneliness resulting from the COVID-19 pandemic and associated restrictions may increase self-harm and suicidal thoughts and behaviors [37]. Consequently, cards allowing participants to document the impact of new factors emerging since 2013 may be important additions. Furthermore, some cards may need rewording or updating to incorporate changing social views and our understanding of self-harm. The National Institute for Health and Care Research in the United Kingdom emphasizes patient participation and involvement in all funded research. Also, the continued development of CaTS in collaboration with people with lived experience is recommended by other researchers using CaTS [26].

### Adapting CaTS for High-Risk Groups—LGBTQIA+ People

There is increasing awareness that lesbian, gay, bisexual, transgender, queer, intersex, asexual, and other minoritized gender and sexual identities (LGBTQIA+ [38]) people are at increased risk compared to their cisgender (someone whose gender identity aligns with their birth-assigned gender and body [39]) and heterosexual [40,41] peers. Lifetime LGBTQIA+ self-harm prevalence estimates between 49.5% and 65.35% have been reported [42,43]. High prevalence rates demonstrate the need for meaningful research tools capable of examining and identifying antecedents to LGBTQIA+ self-harm. However, many tools and measures used to examine LGBTQIA+ mental health are often cis- and heterocentered and may not capture LGBTQIA+ experiences adequately [44-46]. Other researchers have sought to adapt CaTS for other high-risk populations, including autistic people [47]. Co-producing self-harm tools and measures alongside people with lived experience is highly valuable and recommended by researchers in the field [45,46,48]. Considering the increased risk LGBTQIA+ people face [40,41], it is important to identify whether CaTS requires amendments or additions to ensure it contains meaningful, relevant factors for people who identify as LGBTQIA+.

### Research Aims

Here, we present 2 studies conducted with people with lived experience. Study 1 aimed to pilot a web-app version of CaTS and assess its feasibility and usability. This is essential to establish before use in large-scale studies because poor feasibility and usability reduce engagement and may result in attrition [49]. We also present iterative prototype feedback that focuses on performance, design, and content (ie, whether items or cards needed adding or rewording) and findings from visual analog data examining participants’ mood pre- and poststudy. The latter is important to ensure taking part in self-harm studies using CaTS-D does not adversely impact participants’ mood. Study 2 presents findings from a qualitative interview study that captured amendments and additions that inform the development of the CaTS/CaTS-D. These are important to ensure CaTS remains a useful, meaningful research tool as our understanding of self-harm evolves.

## Study 1—The CaTS-D: Pilot Study

**Figure 1.**
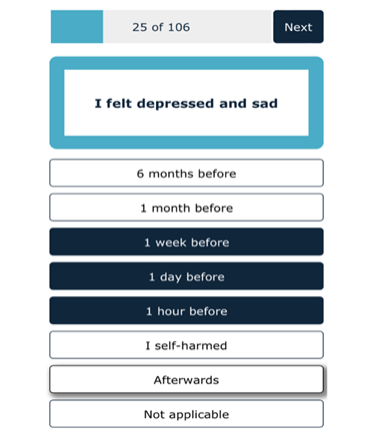
An example of a card presented in Card Sort Task for Self-Harm—Digital (CaTS-D) with several time points highlighted.

CaTS-D was developed using react.js, a JavaScript library capable of developing modular user interfaces (ReactJS [50]). CaTS-D is accessed via a link or QR code, and it was primarily designed to optimize smartphone usage with a vertical layout allowing people to complete the task at their own pace and convenience. Performing CaTS-D involves participants working through cards individually. Each card has all timepoints underneath, and participants select all relevant timepoints for each card before moving to the next card (see Figure 1 for an example card presented in CaTS-D with selected timepoints). Participants select “not applicable” to cards not relevant to their most recent self-harm episode (see Figure 1). It is not possible to simultaneously select timepoints and “not applicable.” Participants work through all cards in this manner until the task is complete. There is an opportunity at the end to manually add up to 4 cards not already included in the task. Here, we present findings from the pilot study that assess the usability and feasibility of the web application CaTS-D. There is also potential for amendments and updates from this pilot to improve user experience, increase task completion, and reduce attrition. To identify these amendments and updates, we captured iterative prototype feedback which focuses on performance, design, and content (ie, whether items or cards needed adding or rewording). Finally, we also present feasibility data and findings from visual analog data. This is important to ensure participating in self-harm studies using CaTS-D does not adversely impact participants’ mood.

### Aim

The pilot study aimed to establish the feasibility and usability of CaTS-D and assess the impact on participants' mood.

### Methods

#### Participants

UK-based individuals with lived self-harm experience, internet access, and a smartphone, tablet, or desktop, and aged 18 years and older were invited to participate via email and social media advertisements through gatekeepers (mental health organizations). Participants were included on the basis of self-reported self-harm history.

#### Ethical Considerations

Participants read the study information and provided informed consent afterwards. Data were stored on a secure University of Nottingham database and will be kept for 5 years. All pilot data is anonymous at the point of participation. Qualitative data was anonymized during thematic analysis and identifying information redacted. Each participant chose their own pseudonym. Participants received a £10 (US $13.45) Amazon voucher remuneration. This research adhered to and was approved by the University of Nottingham’s School of Psychology Ethics Committee (S1543R).

#### Study Procedure

Participants followed the link provided by the research team, which took them to the web application, CaTS-D. Study information and instructions on how to complete CaTS-D were presented first. Participants provided informed consent by clicking a button at the bottom of the page. A unique identifier was provided at this stage, and participants were advised to email author KB with the identifier should they wish to withdraw data. Then, participants completed a brief well-being plan, which involved participants confirming they had details of a trusted person to hand should they need it, and to undertake a poststudy activity to improve mood. Details of support organizations were presented, and participants were advised these would be provided again afterwards.

Participants were asked for their demographic information (age range, sexuality, gender identity, sex assigned at birth, ethnicity, current employment or education status, household income, and education level). Data was collected between November 2023 and January 2024. All data, including consent, were captured during a single session per participant. In line with other self-harm studies (ie, [5,26]), participants completed CaTS-D while reflecting on their most recent self-harm episode. Participants selected all timepoints relevant to that card, or “not applicable.” They worked through cards until the task was complete. To control for order effects, cards were randomly presented each time [51,52]. However, 7 cards relate to what occurred post self-harm; these cards were presented after all other cards. Response options for post self-harm cards were “yes,” “no,” and “not applicable.” Only a single option could be selected. Participants were able to free-type up to 4 items missing from the task and select relevant timepoints, similar to blank cards included in the in-person variant of the task.

Afterwards, to assess feasibility, participants were asked questions based on the System Usability Scale (SUS; [27]). This scale evaluates overall usability and acceptability of technical products including software, hardware, and websites [53]. Questions focused on whether instructions were easy to follow, how participants found completing CaTS-D on their device, and whether CaTS-D was easy or difficult to complete (see Section S2 in Multimedia Appendix 1 for the SUS questions). We used a marginally revised SUS with some items reworded to increase relevance to CaTS-D (ie, changing “system” to “web-app”). An open-ended question requesting further feedback was included to elicit additional comments after each question, and again at the end. Questions in the SUS required dichotomous or Likert-scale responses. Questions 1‐3 captured dichotomous (yes or no) responses, those selecting “no” could also free-type feedback. The remaining 12 items were scored on a 5-point Likert scale (ranging from 1 [strongly disagree] to 5 [strongly agree]). To monitor the emotional impact of using CaTS-D, participants rated their emotional state at the beginning and end of the study by completing a visual analog scale (VAS). This was presented as a scale ranging from 0 (illustrated with a sad face) to 10 (illustrated as a smiling face). Participants were prompted: “Please rate your current mood on the scale below.” Pre- and poststudy VAS data were analyzed to identify whether participating in the study impacted mood. Participants were signposted to support organizations after the study and presented with cute animal pictures as part of debriefing. This presentation aligns with previous CaTS studies [26] and recommended good practice to mitigate mood when conducting sensitive research [54].

### Results

#### Data Analysis

Data was downloaded as a .CSV file, transported into a Microsoft Excel spreadsheet, and analyzed using IBM SPSS (version 28.0). Descriptive statistics regarding participant characteristics were analyzed to characterize feasibility and engagement [55]. A general inductive approach was used to analyze qualitative responses for feedback on CaTS-D regarding whether concepts or cards were missing or needed updating [56].

#### Participant Characteristics

A total of 13 participants participated, and their ages ranged between 18 and 30 years. Demographic data is presented in Table 1. On average, participants spent a mean 16.32 (SD 12.12) minutes completing the task. This is reassuring considering the ideal length of time spent completing online studies is ~20 minutes [57].

The mean prestudy score on the emotional VAS was 7 (SD 1.25) and 5.89 (SD 0.52) afterwards. Therefore, scores persisted around the midrange of the scale (1-10) before and after completion. A paired-samples *t* test was conducted to compare pre- and poststudy VAS scores. This did not represent a significant difference (*t*_9_=1.59; *P*=.15).

**Table 1.** Pilot study demographic data^a^.

Demographic characteristics^b^	Participants (N=13), n (%)
Ethnicity	
White British	9 (69)
White other	1 (8)
British Asian	1 (8)
British Chinese	1 (8)
British Indian	1 (8)
Gender identity	
Cis female	9 (69)
Cis male	2 (15)
Nonbinary	2 (15)
Birth-assigned sex	
Assigned female at birth	10 (77)
Assigned male at birth	2 (15)
Prefer not to say	1 (8)
Sexuality	
Heterosexual	5 (38)
Bisexual	4 (31)
Other	4 (31)
Marital status	
Single or never married	12 (92)
Married	1 (8)
Education level	
Bachelor’s degree	3 (23)
College or university—no degree	6 (46)
PhD or doctoral degree	3 (23)
A- or T-levels	1 (8)
Employment status	
Employed full-time	4 (29)
Employed part-time	4 (31)
Unemployed	4 (31)
Prefer not to say	1 (8)
Household income	
£40,000-£49,000 (US $53,783-US $65,884)	2 (15)
£50,000-£59,000 (US $67,233-US $70,330)	1 (8)
£60,000-£69,000 (US $80,675 -US $92,776)	1 (8)
£70,000-£79,000 (US $94,120-US $106,222)	2 (15)
£100,000+ (US $134,464+)	1 (8)
Prefer not to say	5 (38)

a The options used to capture the demographic data in this study were in accord with demographic data captured by the Office for National Statistics or UK government studies [58].

b Demographic categories not selected by any participants are omitted.

#### Feasibility of CaTS-D

The maximum mean score for all SUS subscales was 5. Higher scores indicate greater perceived usability [53]; therefore, a mean score of 4 was selected as a test value. This is in line with other pilot studies which used the SUS to assess feasibility of self-harm and suicide interventions in smartphone applications [53]. Mean scores on the SUS subscale ranged from 3 to 5 (mean 4.65, SD 0.53). However, the mean response for Q10 (“I think that I would like to use this web-app frequently”) was 3. This represented “neither agree nor disagree” that was an appropriate response considering the nature of the web application for single-use research purposes. Across all 15 SUS questions, there were some nonresponders. High scores on remaining questions are reassuring and indicate participants will not struggle to complete CaTS-D and should complete the task competently and efficiently. Considering the recorded mean was higher than the test score (4), statistical tests were not run.

#### Qualitative Feedback

Participants were given the opportunity to provide written feedback on all SUS questions. Overall, participant responses suggested pre-study instructions should be made clearer. Responses were provided for three questions (“Were instructions easy to follow?” “Are cards visually appealing?” “Did the instructions make completing the task easy?”). Feedback reported by participants included:

- “I wouldn’t say they were difficult, but there were a few things that I missed originally, such as it being related to my most recent occasion of self-harm.”
- “The timeline questions were a bit confusing, and I wasn’t always sure how ‘I self-harmed’ matched up with the card. For some things it was just happening basically always.”
- “I was not sure when I had to tick the ‘I self-harmed’ box”

We recommend researchers ensure instructions are clear when using CaTS-D in research, including which self-episode the research relates to, that participants can select multiple or all timepoints, and to explicitly state “I self-harmed” should be selected if factors are relevant during the moment of self-harm (a copy of the instructions we used is available upon request).

### Discussion

#### Principal Findings

This pilot study found CaTS-D is a highly usable, feasible, and acceptable research tool and participation did not negatively impact participants’ mood. The study aimed to assess the usability, feasibility, and acceptability of CaTS-D and capture iterative feedback of CaTS-D in order to identify amendments to increase the usability of this novel web application. Assessing usability, feasibility, and acceptability is an essential part of web-app development to ensure the web application is easy to use and complete in order to reduce attrition [49]. High scores on the SUS were endorsed by participants across all items. Responses to SUS questions indicate participants found CaTS-D consistent, easy to use, and easy to perform. Furthermore, participants felt confident using CaTS-D, did not feel CaTS-D was unnecessarily complex, and well-integrated. Regarding card design, feasibility data showed participants felt the font and card interface were easy to read and visually appealing. In addition, participants felt they would not need technical support or learn anything to use the web application. Considering the nature of online research, this is reassuring and indicates future participants will be able to complete CaTS-D remotely without difficulty. Finally, although VAS scores decreased slightly poststudy, statistically, participants’ emotional state was not negatively affected by completing CaTS-D. However, our sample size may be too small to determine whether this reduction was statistically meaningful. A slight mood reduction may be due to the effects of thinking about self-harm experiences. Indeed, previous CaTS research noted mixed results with VAS reductions in mood shown for younger people but not for adults [26]. However, there is no evidence that partaking in self-harm research has significant negative impact on mood [59]. This is reassuring, but further studies with larger samples can confirm whether our sample was too small (low power) to detect an effect. Importantly, the positive mood induction at the end of the study ensures participants will not be negatively impacted by taking part in studies using CaTS-D.

The large number of nonresponders to the open-ended feasibility questions is disappointing. This may suggest participants were fatigued having responded to each card and completed the CaTS-D immediately before answering SUS questions. Indeed, response burden is associated with reduced attrition and unwillingness to engage in research [33]. Furthermore, some nonresponses may be due to the wording of particular questions. For example, Q6 whether “various functions of the web-app were well integrated.” Possibly, participants were unsure how this concept related to CaTS-D or did not understand “integrated” in this context, so chose not to respond. Wording this question differently may have yielded more responses. In hindsight, it may also have been prudent to ensure participants could not continue without responding to feasibility questions. Though this may have meant participants felt forced to provide responses, this may result in attrition and fewer overall responses being provided. Despite this, however, feedback and responses provided were positive and indicate CaTS-D is easy to use, does not require additional learning or support to complete, and people feel comfortable completing CaTS-D. The pilot data presented here suggests CaTS-D is a promising, feasible, acceptable, and useful web application for use in self-harm and suicide-related research. It can be used to examine and identify key self-harm antecedents, which can be targeted during intervention, if present.

Qualitative feedback was brief and suggested instructions could be clearer. Based on this feedback, we make the following recommendations for researchers using CaTS in future research:

- Instructions should explicitly highlight which self-harm episode (ie, first ever, most recent) should be considered by participants taking part in the study
- Ensure instructions clearly explain “I self-harmed” is a timepoint that can be selected if cards are relevant to the point of self-harm
- To explicitly state all timepoints (including “I self-harmed”) can be selected if necessary.

Following these recommendations will reduce participant confusion and ensure data captured in future studies directly relates to the research question.

#### Strengths and Limitations

Participant demographics included a range of employment statuses, education levels, and household incomes indicating the acceptability of CaTS-D in a wide population. Similarly, most participants (~70%) were White British. While this may seem an inflated figure, it is lower than recently reported population figures from the Office of National Statistics (81%; ONS [60]. Importantly, the sample included substantial representation from LGBTQIA+ communities (n=2, 15% nonbinary and n=5, 62% nonheterosexual) aligning with the higher prevalence of self-harm reported in these groups. Therefore, the pilot study sample is largely in line with, thus representative of, the UK population. However, we recommend further feasibility testing to ensure feasibility in other countries.

#### Conclusion

These findings indicate participants can complete CaTS-D easily and confidently. Using CaTS-D addresses some issues faced by researchers, such as gathering data from hard-to-reach populations [34]. Furthermore, the anonymity CaTS-D offers may be preferable, including people in hard-to-reach populations. Considering most people use smartphones and devices daily [29,31], tools developed for online mental health research are increasingly necessary and, in some instances, preferable to in-person research [53,61]. While many people quickly navigate new websites or web applications [62], accessibility and efficiency are essential [49]. Consequently, assessing the feasibility of newly developed web applications is an important, necessary step. CaTS-D is a novel, unique research tool allowing participants to document their bespoke self-harm pathway. Results from this pilot study establish CaTS-D as a feasible, usable research tool, suitable for capturing large-scale self-harm data.

## Study 2—Co-Developing Updates to the CaTS With Young People With Lived Self-Harm Experience: Qualitative Interview Study

Since CaTS was developed in 2013, factors may have emerged which need including to increase the relevance and appropriateness of CaTS. In addition, cards may need rewording or updating to incorporate changing social views and our understanding of self-harm. Furthermore, considering LGBTQIA+ people are at increased risk of self-harm, it is important to know if and what needs adapting for people who identify as LGBTQIA+ to ensure CaTS is suitable to fully capture LGBTQIA+ self-harm experiences.

### Study Aims

We asked cisgender-heterosexual and LGBTQIA+ people with a history of self-harm for their views on CaTS. These included the relevance of cards, the timeline, clarity, language, and wording on cards, and whether participants felt amendments or additions to CaTS were needed. We also asked whether LGBTQIA+-specific additions were needed to allow LGBTQIA+ people to best document their self-harm pathway using CaTS. This study will ensure CaTS is contemporary, sensitive, and appropriate for use in a diverse population, which is essential for CaTS to remain a useful, meaningful, and inclusive self-harm research tool.

### Methods

#### Recruitment

Semistructured one-on-one interviews were conducted on Zoom with UK residents aged 18 years and older with self-reported self-harm and access to a tablet, smartphone, or desktop or laptop. Participants were recruited from the same organizations as study 1; therefore, some may have taken part in both studies. The anonymity of study 1 prevents us from determining whether any participants also took part in study 2. However, some interviews took place before recruitment for study 1 commenced.

#### Ethical Considerations

Ethical approval was received from the University of Nottingham’s Ethics Committee (S1557). Participants were recruited via social media and via existing relationships with relevant organizations (ie, mental health charities; LGBTQIA+ organizations). Because we aimed to capture views from both LGBTQIA+ and general populations, advertising efforts targeted both groups. Participants expressed interest by contacting the lead researcher (KB) via email, who emailed them full study information, consent form, and a well-being plan. Participants were encouraged to ask questions before providing informed consent and subsequent interviews. The well-being plan ensured participants were supported before and after participation and detailed that safeguarding concerns would be disclosed to relevant parties should they arise. The plan provides information on support organizations and details what would occur should participants become distressed during and after the study. The well-being plan required participants to detail their poststudy activities and provide details of a trusted person, their general practitioner, and the address they will be at during the interview in the event they become distressed during the study and researchers needed to call an ambulance. A copy of the Wellbeing Plan is available in Section S13 in Multimedia Appendix 1. Once participants completed and returned consent forms and well-being plans, a mutually convenient meeting was organized using a Zoom or MS Teams link, and a PowerPoint document containing the original CaTS (see Section S1 in Multimedia Appendix 1) was emailed to participants to consider before the interview.

#### Procedure and Data Analysis

Interviews took place via between June 2023 and February 2024. Research shows that interviews that took place via Zoom elicit comparable participant-researcher rapport to in-person interviews, are a similar length, and are popular with participants [63]. The interview schedule explored participants’ views of existing CaTS cards and concepts, the timeline, language, clarity, and wording on cards, and whether general population or LGBTQIA+-specific additions and amendments were necessary to ensure CaTS contains meaningful, relevant factors. Before interviews, participants provided verbal confirmation they had read and understood study information and felt comfortable taking part. Participants were not primed by topics raised by previous participants to avoid influencing their responses, ensuring themes identified through subsequent thematic analysis reflected participants' independent perspectives. Interviews were undertaken by KB, lasted between 40 minutes and 1.5 hours, and were audio and video recorded. Interviews were manually transcribed verbatim and checked by a member of the research team. All transcripts were anonymized using pseudonyms chosen by each participant. Afterwards, participants were asked if their emotional state had changed during the study and additional debrief information, including signposting to support services and organizations, was emailed to participants. Interviews were followed by a pre-arranged call 24 hours post study to ensure participants’ mood had not been negatively impacted by taking part. Participants received a £10 (US $13.45) Amazon voucher as remuneration for their time.

We used thematic analysis to identify themes in our qualitative dataset. This approach was ideal compared to other qualitative analysis methods because it is not underpinned by epistemological or theoretical frameworks [64]. The flexibility of thematic analysis allowed us to generate codes and identify key features within the data to inform amendments and additions to CaTS variants. We performed Thematic Analysis by completing its six phases: (1) familiarization with data via transcription, (2) systematically generating initial themes, (3) searching for themes, (4) reviewing themes, (5) defining and naming themes, and (6) producing the final report. Final transcripts were uploaded to NVivo (version 9; Lumivero) for initial coding by KB (phase 2). Initial coded themes were then used for overarching theme analysis. This was performed independently by KB and a member of the research team who resolved discrepancies in interpretations via discussion.

### Results

Demographic information is presented in Table 2. Participants (N=13) aged between 21 and 29 years (mean 24.67, SD 4.95) from the United Kingdom were interviewed. A total of 9 participants were LGBTQIA+ (mean age 23.56, SD 3.24 y) and 4 were cisgender heterosexual participants (mean age 23.75, SD 3.59 y). Participant characteristics varied, ensuring wide representation in the LGBTQIA+ community. Data including potentially identifiable information (ie, location) was omitted from quoted extracts to ensure confidentiality.

**Table 2.** Demographic characteristics of participants (Interview study)^a^.

Demographic characteristics^b^	Participants (N=13), n (%)
Sexual orientation	
Bisexual	6 (46)
Lesbian	1 (8)
Queer	1 (8)
Pansexual	1 (8)
Heterosexual	3 (23)
Gender identity	
Cisgender female	9 (69)
Cisgender male	2 (15)
Nonbinary	1 (8)
Questioning	1 (8)
Ethnicity	
Chinese	1 (8)
White British	10 (77)
Mixed (White and African)	1 (8)
Mixed (White and Asian)	1 (8)

a The options used to capture the demographic data in this study were in accord with demographic data captured by the Office for National Statistics/UK government studies [58].

b Demographic categories not selected by any participants are omitted.

Initial questions focused on the clarity and relevance of wording on cards. Here, data was descriptive and explicit and is presented as such. Thematic analysis identified themes resulting in the creation of 13 new cards and amendments to wording on specific cards. Additional suggestions included capturing neurodiversity data and emphasizing sexual violence trigger warnings. Participants provided themes for cards and most offered literal suggestions regarding how cards could be worded.

#### Language, Terminology, and Minor Updates

##### Update Terminology

Overall, participants felt the cards were clear and relevant to self-harm; however, some felt terminology should be updated. This related specifically to cards, including “forum.” Coral explains:

…there was like these two cards around….‘I read about self-harm on the Internet’ and ‘I discussed self-harm in a forum’ … I don't know if it’s a little bit like old-school…maybe changing it to social media or something like that because…like although some people still use forums, I don't know if that’s totally relevant or not.

Others also felt “forum” should be replaced with “social media/the internet” to reflect changing internet habits (see SM3 for additional quotes). Consequently, cards containing “forum” will be updated to “social media/the internet” to reflect changing internet usage and associated terminology. In addition, several participants suggested updating cards using “girlfriend/boyfriend” to a gender-neutral term. Coral explains gender-neutral language is necessary to ensure inclusiveness:

…there was one here ‘I had an argument with my boyfriend/girlfriend’. I think that should just be changed to ‘partner’, so it’s a bit more inclusive…

Others agreed the word “partner” would capture all romantic relationships, regardless of your sexuality or gender expression (see Section S4 in Multimedia Appendix 1 for further quotes). Changing cards worded “girlfriend/boyfriend” will ensure they are relevant to all romantic relationships, regardless of marital status, gender expression, or sexuality. Resultantly, these cards will be updated to reflect changing discourse around inclusivity.

### Capture Neurodiversity Data

While neurodiversity was not the study focus, or specifically asked, several participants discussed the overlap between neurodiversity and LGBTQIA+ identities. Participants felt capturing neurodiversity alongside demographic data would be useful. For example, Bronwyn said:

There’s obviously the big overlap with LGBTQIA and neurodiversity, and I'm not sure how much of neurodiversity is completely captured. Maybe it could be something that is talked about before the task…if you're gonna get some demographics beforehand, can they just type in or like click a button that says like, Yep, this neurodiversity…people tend to want to say ‘by the way I have this sort of neurodiversity’. So, they want to kind of explain why they're saying things in a certain way

Other participants highlighted the LGBTQIA+ and neurodiversity overlap, and how neurodiverse people like the option of explaining or discussing their neurodiversity (see Section S5 in Multimedia Appendix 1). Consequently, we recommend researchers capture neurodiversity alongside sociodemographic data to allow participants to describe this important aspect of their journey.

### Highlight Sexual Violence Trigger Warnings

Some cards relate to sexual violence. The introductory information contains a trigger warning to alert participants to this. However, a participant, NE, felt this should be more significant:

…Luckily I don't have experience with that but I know people who do and I feel like if they were doing this card sort, that would have been very…triggering for them…But when I was doing the pilot study…it just feels like it comes out of nowhere, which can be quite triggering for some people… there was almost definitely was a sexual assault warning before the study. But I think emphasising that would be very good…

While only a single participant highlighted this, ensuring trigger warnings are clear and explicit will ensure participants are forewarned some cards contain terms relating to violence and sexual violence. Evidence regarding the effectiveness of trigger warnings in reducing distress is mixed [65,66]. However, trigger warnings do not lead to task avoidance [67], and evidence suggests people expect and support trigger warning use in research to forewarn people of potentially traumatic content [65,66]. Consequently, we recommend researchers present content and trigger warnings in recruitment and consent literature regarding content relating to potentially traumatic events.

### Timeline—Additional ‘Before 6-Months’ Timepoint

Overall, participants felt the existing timeline is appropriate to capture self-harm factors at specific times pre– and post–self-harm. However, 9 participants felt an additional timepoint to capture historic events would be beneficial to examine why self-harm began, or to document a particular historic event occurred which links to other factors. Below we present two 2 quotes but see Section S6 in Multimedia Appendix 1 for further quotes. Selma explains:

…some people will…develop, like, self-injurious patterns really early on. Like, I know it was the case for me…and that is relevant in how there’s self-injury…I think, you know, maybe you could have, like, a card that says, more like ‘historical’, but maybe in, like, a different wording…

Here, Selma discusses why historic factors are important to document when considering one’s self-harm pathway:

…there’s like ‘I was abused’…ermm….so I think that’s the case for a lot of people who self-harm…but a lot of the time is more historical…so people have that in their history. It might not be relevant, like directly relevant, to their most recent episode of self-harm, but it might contribute to why they started self-harming in the first place…

Together, the quotes indicate participants feel capturing historic events with an additional “before 6-months” timepoint is important. Participants discussed the longitudinal nature of self-harm and the association between historic risk factors and subsequent self-harm and how an additional “before 6-months” timepoint would allow researchers to capture important data. Other reasons for including an earlier timepoint focused on significant childhood or historic events, particularly physical or sexual violence (see Section S6 in Multimedia Appendix 1 for further quotes). Participants felt documenting historic events occurring before 6 months, before a self-harm episode, will provide deeper understanding. In particular, traumatic events that may result in self-harm and other related risk factors. Therefore, a card entitled “Before 6-months” will be added to ensure CaTS captures historic events which impact later self-harm and related factors is an important addition.

### Additional Cards

Participants were asked whether cards or concepts were missing from CaTS. Thematic analysis identified themes which provided evidence for the creation of 12 new cards.

#### “I Did Not Feel Comfortable in My Body”

Participants recognized not feeling comfortable in their body as a risk factor. A total of 2 participants explicitly related this to gender dysphoria, but remaining participants discussed how general body image issues are a self-harm factor. The overarching theme encompassed not feeling comfortable in one’s body, and participants felt CaTS should include a card relating to this. Here, James Hogg discusses how not feeling comfortable in one’s body develops for different reasons:

…something to do with body image or feeling as if your body is not right…self-harm’s very common with people with body dysmorphia, but also you know a lot of body dysmorphia comes with gender identity and stuff like that…being bullied can also cause you to have issues with your body as well, that you didn't have before…I think it’s a very common thing that people who have self-harm experience that isn't currently covered and it also just by coincidence also links quite heavily to the LGBTQ-plus community.

CH agrees a card incorporating body image issues would be useful for both LGBTQIA+ and non-LGBTQIA+ people:

…maybe there could be a card for like for dysphoria…like a ‘I don’t feel…that my body is correct’ or like ‘I, I hate my body’ card or something, because I feel like that’s general enough to apply to non-LGBT people, but then like I know body dysphoria is like a big thing for trans people.

Similarly, NE agrees a card documenting body-image issues would apply to people in different communities for various reasons:

…there’s no cards on like especially for transness. I am nonbinary, so…I feel like I can contribute a lot in this area, but there’s no cards on…gender dysphoria, or just general body dysmorphia, which I think could contribute even for…non-LGBT people…

Together these quotes (and others in Section S7 in Multimedia Appendix 1) indicate CaTS requires a card allowing participants to document body image issues as a self-harm risk factor. This aligns with emerging literature showing body image issues are associated with self-harm [68]. Body image issues are also associated with general psychopathology (eg, depression and anxiety [69]). Depression and anxiety are well-evidenced factors for self-harm [70] and already included in CaTS. Possibly, a temporal relationship between body-image issues and other factors exists which increases self-harm risk. Including a card relating to body image issues would ensure CaTS can examine this pathway. Many participants explicitly discuss gender dysphoria and body dysmorphia; however, participants using CaTS may not have received specific diagnoses, nor feel their experiences fit into diagnostic categories. Including a card that incorporates body discomfort more generally will appeal to more participants than cards applying to specific body image-related diagnoses. The term “I did not feel comfortable in my body” was selected because feeling comfortable in one’s body is associated with body-image issues and is a phrase used in measures of body appreciation (eg, Body Appreciation Scale-II [71]). Furthermore, it is sufficiently broad to be relevant for various body-image issues.

#### “I Felt I Did Not Fit In”

Participants discussed feeling not fitting in with wider society, or sometimes their own community, and this relates to both LGBTQIA+ people and the general population. Here, NE describes conflict within the LGBTQIA+ community can make people feel they do not fit in with people they should fit in with:

…I would say there’s quite a lot of infighting in the community which I think could severely isolate a person. I know there’s a card on ‘I felt isolated’…But maybe having like another card that’s… something to do with I felt excluded from people who should…people I should be fitting in with or people I, I thought I could trust…

Similarly, James Hogg discussed in-fighting within the LGBTQIA+ community can result in people feeling they do not fit in, and this may lead to self-harm:

…you've got the whole LGBTQ umbrella. But then within that each one of those letters in that acronym will have different issues related to their sexual identity or their gender identity. So, you know, there’s infighting in the trans community, there’s infighting between bisexual people and homosexual people, you know…there wasn't a question that related to the feeling of not fitting in…with queer people there’s obviously the idea of not fitting in with the straight people…but then also within the community itself, as I said, there’s infighting. There’s rejection within the community because you're not properly trans, you're not properly gay, you're not properly whatever. So, I feel like…the feeling of not fitting in drives a lot of people to isolation and therefore self-harm.

While NE and James Hogg discuss not fitting in from an LGBTQIA+ perspective, the concept may impact people from various sociodemographic backgrounds. Coral highlights this:

…I think there was another one…maybe like ‘I felt like I didn't belong in my community’. I think that could be quite…a big one, especially if there’s like a religious community or culture or something that I guess could be related.

Here, Coral talks about how people from different social groups can feel they do not fit into their community, which could be a factor for self-harm. These quotes identify “not fitting in” as an important factor (see Section S8 in Multimedia Appendix 1 for further quotes). Not fitting in is strongly associated with isolation [72], which is already captured within CaTS. However, not fitting in may precede isolation. Indeed, James Hogg states this at the end of his quote: “the feeling of not fitting in drives a lot of people to isolation and therefore self-harm.” Adding a “not fitting in” card will allow participants to document this concept in their pathway and ensures CaTS can examine temporal relationships between not fitting in, isolation, and self-harm.

#### “I Found Support/a Community on Social Media” and “Social Media/Online Content Was Negatively Impacting My Mental Health”

Participants discussed the positive and negative impact of social media and online content on self-harm. First, Jo talks about the support people can find online:

…you’ve also got the flip side of people’s self-harm, I think is particularly common at finding communities online, especially if you don’t kind of feel accepted or have a place in the real world. I guess then, I think social media can actually be somewhat of a safe haven as well because you can present yourself differently or you can like share stuff easier with strangers online than you can face to face with people that you actually know. So, I think, yeah, obviously it can be toxic, and it can be a scary place. But I think for people who are struggling, it can also be quite a helpful place…it could be like…I reached out online which helped' or I yeah, 'I received peer support online and found a community online' or something like that because I think online support is missing from those [cards]…

Here, Jo discusses how social media can provide solace, community, and a sense of belonging, especially if one lacks this in the “real world.” She acknowledges social media can be toxic, but she clearly feels the positive effects of social media interaction should be documented within CaTS. Belle also highlights how online self-harm groups can provide a sense of community and belonging:

…people are doing this too like ‘that’s nice’. Like I’m not the only one doing it. And then you feel like you can compare and almost not do it with them in some way, even though it’s your own pain, it’s not the same…You're not experiencing the same thing, but you make it feel like it’s an altogether group thing.

Seeing self-harm content online may engender a sense of community and belonging. However, NE discusses how social media can negatively impact mental health:

…I feel like younger kids, like 13,14 social media is gonna be a huge impact on your mental health…If you're self-harming…that probably would be good to mention something about. I’m not sure what specific cards you would have…something mentioning about how…social media is impacting your mental health, or you know, like seeing if…other people are clearly doing much better in life…

Here, NE discusses the impact that comparing yourself negatively with others on social media can have on mental health and self-harm, particularly in younger people. Others agree (see Section S9 in Multimedia Appendix 1 for further quotes), and some made suggestions on how a card could be worded. For example, Artemis suggested: “...just a generic ‘I felt like social media was impacting my mental health’...it would mean different things to different people, but it would be kind of a useful catch all.....”

CaTS was developed before the significant rise in social media and smartphone usage that occurred over the past decade [73], so the original version does not contain social media-related cards. The quotes presented here highlight the significant presence of social media in contemporary life and its potential impact on self-harm. The positive and negative impact social media has on self-harm has been documented by others (ie, [35,73]), and participants clearly feel cards allowing users to document social media experiences and its impact on self-harm are important additions.

#### “I Was Bullied on Social Media” and “I Was Discriminated Against on Social Media”

Alongside the potentially negative impact of social media on self-harm, participants talked specifically about being bullied or discriminated against online being a factor for self-harm. Artemis says:

…social media…it’s more sort of a tool that kind of exaggerates things in your life rather than a standalone thing. So, you know, if you’re talking to friends, it allows you to talk to them 24/7 rather than just when you're with them or if you’re taking, you know, pictures, it allows you to send them to all your friends rather than…one by one. So, it…allows you to do things on…a bigger and quicker scale. So, it’s sort of…initial things are kind of already encompassed by the cards…so maybe ‘I was bullied’ would include bullied over social media or bullied as a result of social media ‘cause…it would be exacerbated by social media…

Here, Artemis says social media is an extension of one’s life and allows things to occur “on a bigger and quicker scale,” rather than being a separate experience. They state bullying occurs online and on social media as well as in person, and CaTS needs cards which capture online experiences. CS agrees and feels CaTS should also include online discriminatory experiences:

…something…that’s sort of social media. It could look around things like cyberbullying, or things like…sexual harassment on…Facebook or Instagram, or even like something like, I don’t know…homophobic abuse on Twitter that someone may, might have, like, maybe responded to you on there…anything like that would be I think, quite relevant

The narratives here underline bullying and discrimination that occurs in person and online. Clearly, participants feel CaTS should provide opportunities to document whether online bullying or discrimination precedes self-harm. Possibly, other phenomena may occur in dual settings. Future research should explore whether other factors occur online and in-person, and whether additional cards should explicitly refer to factors occurring online.

#### “I Had an Upsetting Sexual Experience” and “I Was Sexually Abused or Assaulted”

Selma talked at length about the “I was raped” card, and how some people may be reticent to select that card, even though they had experienced rape, but may select a card describing unsettling sexual experiences. Selma also felt CaTS should include a card relating to being the victim of sexual assault:

…there’s one card that says ‘I was raped.’ And I think a lot of people, even if it did happen to them, they’re not going to pick it because just reconciling with that term takes a lot of, like, inner work…I think a lot of people, they would not, especially if they’re…in the midst of their healing journey, they’re not going to pick that card, even if it’s relevant to them. I think there needs to be a bit like more nuanced like, like an ‘upsetting, like sexual experience’… I don’t think a lot of young women like, would outright say, ‘oh, I was raped’… things are changing culturally, and people are using that term more freely, but there’s still a lot of stigma…Whereas if you say, oh, you know, ‘I had, like, a upsetting sexual experience’, I’m not even saying sexually abused because even…implying abuse, I think for some people it’s even too much…even though… sometimes clearcut rape or abuse does occur. But it takes the victim a long time to…reconciliate with the fact that it happened…they wouldn’t pick that card even though it might have actually objectively happened, you know?

Here, Selma talks at length about 2 things. First, she highlights that rape does not always occur, but unsettling sexual experiences may occur and be a significant factor. Second, Selma feels people may not reconcile their experience with the “I was raped” card. A card allowing people to document unsettling sexual experiences would address these issues and be used in lieu of the “rape” card if they feel unable to select that card or relate rape to their experience. Others agreed a card covering sexual abuse and assault is necessary to include (see Section S10 in Multimedia Appendix 1 for additional comments). The narrative presented shows CaTS should include cards relating to both sexual abuse and assault and unsettling sexual experiences. Furthermore, sexual assault in adults [74] and childhood sexual abuse [75] are known antecedents for self-harm. Therefore, additions to CaTS which explore their impact on self-harm in relation to other factors will provide valuable insight.

#### “I Was Bullied Because of My Sexuality or Gender Identity” and “I Was Discriminated Against Because of My Sexuality or Gender Identity”

LGBTQIA+ participants discussed being bullied and discriminated against and how, for LGBTQIA+people, this occurs for numerous reasons. CaTS currently includes cards documenting generic bullying and discrimination experiences. However, LGBTQIA+ participants felt discriminatory and bullying experiences specific to sexual and gender identity were important to include in CaTS. CS explains:

I think it would be good to have a bit more…LGBT specific…I’m maybe thinking something like…discrimination that someone might face. Or I don't know a card, for example saying, I don’t know, ‘I’ve been bullied or discriminated against because of my sexuality or identity’ or whatever, so something may be more identity specific…bullying…so, it could be maybe a bit more, erm, specific [to their LGBTQ+identity]…

Artemis agrees gender-specific factors are important and discusses how cards that explicitly specify LGBTQ+-specific experiences will encourage people to select cards if relevant:

…if your LGBTQ identity did tie into any kind of episodes of self-harm, so having that there might make you think, oh…was that part of the reason why I ended up self-harming, you know, because I thought it was just because I was feeling, you know, really sad that day. But looking back, the day before…someone had…pointed out something or someone was…being really prejudiced…but I think having said that [a card] explicitly mentions just an LGBTQ+ identity might be quite useful…you could have, you know, ‘I felt discriminated against because of my…’sexual identity, gender identity, trans identity’…sort of almost, yeah, combine a few into the same card…

Zoe also felt LGBTQ+-related specific discrimination or bullying cards may encourage people to select cards relevant to their self-harm:

…because there’s so many ways of discriminating as well. It might be easier to pick it out if it’s prompted, I guess [LGBTQ+ identity]. Because I feel like for me, when I think of discrimination that, like a few that come to mind. So yeah, I guess it could be interesting to explore the different types of discrimination…

Here, Zoe states people may connect the existing “discrimination” card with other discriminatory experiences. Participants highlight how LGBTQIA+ people experience identity-related discrimination and bullying alongside other bullying and discrimination experiences (see Section S11 in Multimedia Appendix 1 for further comments). Therefore, adding LGBTQIA+-specific discrimination and bullying cards will allow participants to document both experiences. These will increase CaTS’ inclusivity and relevance to LGBTQIA+ self-harm and are important considering LGBTQIA+ people are at increased self-harm risk [43] and tools used to examine self-harm are frequently insufficient to capture LGBTQIA+-specific experiences [44]. Furthermore, while LGBTQIA+ people experience greater levels of discrimination and bullying compared to their cis-hetero peers [76], cisgender women also experience elevated levels of gender-based discrimination [77,78]. Therefore, these cards may also be relevant to cis women.

#### “I Was Questioning My Sexuality or Gender Identity”

In addition, LGBTQIA+ participants felt people questioning their sexuality or gender identity needed the opportunity to document this if relevant to their self-harm. Artemis explains:

…maybe in events…erm…things to do with exploring your kind of identity. I was exploring my gender identity, my sexuality…being with someone or by, you know, just sort of thinking about it, kind of reflecting internally…something like having an identity crisis or ‘I was questioning my sexuality and my gender identity’, or yeah, I think there’s, ‘there’s a lot of questioning’ I think just in general and that might be a big factor…

NE agrees questioning one’s identity may be important for some to document:

"…I’m pretty sure there is a card about saying ‘I didn’t understand how I felt’ or something like that…I, I think if you're having an LGBT-specific one either adding another one or just changing the wording of that where it’s, I didn’t understand like myself or my identity or I was struggling with my identity and how I felt or something like that…

Artemis expands this by describing how exploring your identity can trigger feelings of uncertainty around societal acceptance and expectations of others:

…or…say you were sort of with someone and you thought, ‘oh, I think maybe I’m attracted to them’ or ‘I’m not’, you know, ‘quite allowed to be’. ‘I shouldn’t be’ and sort of…kind of event triggering something… like this…isn’t what I should be doing. You know, ‘my family wouldn’t accept that’, ‘I can’t be doing that’…But yeah, having that kind of, yeah, sort of exploring or thinking about or kind of reflecting on some aspect of your identity and not being happy with that might be quite triggering…

The narrative here shows questioning one’s gender identity or sexuality can lead to internal conflict or confusion, and people are concerned how others may react. People questioning their gender identity or sexuality may experience elevated rates of mental health difficulties and self-harm [79]. Therefore, a card has been added allowing people to document questioning one’s sexuality or gender and self-harm (see Section S12 in Multimedia Appendix 1 for additional quotes).

#### “Self-Harm Gave Me a Feeling of Control”

The final theme identified was the sense of control self-harm can elicit. Participants talked of feeling powerless and how self-harm can help people regain some autonomy. Selma explains:

…I thought maybe it would be good to have something ‘cause I know, again for a lot of women who self-harm, it’s sort of a way to regain bodily autonomy, bodily ownership…erm, and I think it could be good to have a term that kind of captures that on the cards like ‘I felt like, I …powerful’… I put ‘ownership’… I can remember some feelings very clearly in this. Yes, this feeling of like ownership of power, of control.

Zoe also talked about how external factors elicit a lack of control and how self-harm can help people regain control:

…I don’t know if this was mentioned as well, but… one of them is like the lack of control…more as a general feeling of lack of control. Yeah, and how, when people’s lives seem to not be in order. Umm, I guess self-harms one of the ways that they try to get control back…

Similarly, Belle agrees self-harm can help people regain control:

…thoughts of like wanting to feel something, not necessarily like…yeah, just wanting to feel, not necessarily even like pain or like feeling upset…It’s just feeling something…and wanting to take control, like a control thing…

The narrative here indicates that people who self-harm may feel they lack control and autonomy. Participants feel self-harm can be used to regain some control, and this may relate to external or internal factors. This is in accord with findings from recent qualitative systematic reviews where participants described self-harm as a mechanism to regain control related to distal, proximal, and internal factors [80,81]. Indeed, regaining control may be an important factor for repeated self-harm. Considering this, a card entitled “Self-harm gave me a feeling of control” has been added to CaTS.

### Discussion

#### Principal Findings

This qualitative study found that participants generally viewed CaTS as clear, relevant, and appropriately worded, while identifying key additions and amendments to improve inclusivity and relevance for both LGBTQIA+ and general populations. The aim of this qualitative interview study was to garner views of people in LGBTQIA+ and general populations about the content of CaTS, whether cards are worded appropriately, and whether important cards are missing that are relevant to both LGBTQIA+ people and people in the general population. This process identified key additions and 3 amendments to improve the relevance of CaTS to ensure it remains a meaningful, relevant self-harm research tool. Based on the research questions and grounded in the data, we clarified that participants felt cards were clearly and appropriately worded, and concepts on cards were largely relevant to self-harm. Resultantly, additions and future cards will be worded following a similar linguistic format. However, participants suggested changes to ensure CaTS uses contemporary and inclusive terminology: changing “forum” to “online/social media” and using the gender-neutral term “partner” where relevant. Furthermore, participants discussed the importance of capturing neurodiversity status. In accordance with extant literature [82,83], participants highlighted links between neurodiversity and LGBTQIA+ identity and expressed the need for neurodiverse people to document their neurodiversity. Capturing neurodiversity data alongside other sociodemographic data would identify neurodiverse-specific self-harm antecedents. Furthermore, researchers may identify links between neurodiversity and self-harm, and exploring this pathway is recommended by experts (eg, [84]). We recommend researchers capture neurodiversity data using categories (eg, Autism, Autism Spectrum Disorder [ASD], Attention Deficit Hyperactivity Disorder [ADHD], dyslexia, dyspraxia, Asperger's, and others) in line with suggestions from service providers who support neurodivergent people (ie, Diversity Project and The Brain Charity). Finally, a participant discussed a clearer, concise trigger warning relating to sexual violence content. Because trigger warnings are commonplace, expected, and people support their use in research [65], we recommend researchers very explicitly state some cards relate to physical and sexual violence during recruitment and informed consent to avoid unnecessary surprise or discomfort.

#### New Cards

The heterogeneity of factorial themes developed from these data mirrors the heterogeneity, complexity, and variety of factors for self-harm experienced. However, thematic analysis identified themes that supported the development of 12 new cards and 1 timeline card. First, analysis showed participants felt CaTS should include a “before 6-months card.” Participants felt this would allow people to document significant childhood or historic events important to their self-harm pathway. This inclusion will enable CaTS to examine historic factors and identify whether specific historic factors are temporally linked to subsequent factors and eventual self-harm. Because participants were aware that the study aimed to identify potentially new cards, most offered suggestions regarding how cards could be worded. While some LGBTQIA+-specific cards were developed from the data, overall, participants (including LGBTQIA+ participants) suggested themes and cards which could be applicable to people from various backgrounds.

The twelve cards created from themes identified within the data are:

1. “I don’t feel comfortable in my body”
2. “I felt I did not fit in”
3. “I found support/a community online”
4. “Social media/online content was negatively impacting my mental health”
5. “I was bullied on social media”
6. “I was discriminated against on social media”
7. “I had an upsetting sexual experience”
8. “I was sexually abused/assaulted”
9. “I was bullied because of my sexuality or gender identity”
10. “I was discriminated against because of my sexuality or gender identity”
11. “I was questioning my sexuality or gender identity”
12. “Self-harm gave me a feeling of control”

Adding these cards ensures CaTS and CaTS-D contains factors relevant to self-harm and remains a useful, meaningful research tool. LGBTQIA+-specific additions are important to increase the inclusiveness of CaTS. This is particularly important considering self-harm prevalence in LGBTQIA+ people is elevated [42,43,85] and tools used to examine mental health in LGBTQIA+ people are often cis-hetero–centered [44,45]. The LGBTQIA+-specific cards added to CaTS will allow people to document whether factors specific to LGBTQIA+ people are key antecedents to self-harm in this high-risk group. These details are important to capture considering researchers largely focus on general risk and protective factors, meaning group-specific factors are poorly researched [86]. Therefore, the impact group-specific factors have on self-harm is not understood. Using CaTS with the additions developed here will address this deficit in understanding by identifying whether self-harm in LGBTQIA+ people is preceded by particular group-specific factors. Future research with LGBTQIA+ people may identify further additions and is recommended to increase the inclusivity and relevance of CaTS. The remaining additional cards may be relevant to people from various backgrounds and for different reasons. Furthermore, they are important additions reflecting changes in what can result in self-harm. For example, the rise in social media usage and the way we use and engage with others online has changed dramatically over the last decade. Without exception, participants in this study discussed the negative impact social media can have on self-harm. This impact is supported by other researchers in the field (ie, [87,88]) who report increases in self-harm risk correlate significantly with increased time spent on social media. However, participants also discussed the benefits of a community and sense of belonging that can be found online. A recent narrative review discussed benefits including being able to communicate authentically with peers online [36], meaning social media may provide solace to some. Findings regarding social media are polarized. However, adding social media-related cards allows participants to document these experiences and ensure CaTS includes cards relating to a concept, which has an evidence-based impact on self-harm behaviors. Their addition increases the relevance of CaTS, ensuring it remains a useful and meaningful research tool to examine self-harm in cis-heterosexual and LGBTQIA+ people and identify key antecedents which can be targeted during intervention. Future research may identify additional cards or support the development of a set of cards specific to a particular population. The study demonstrates the flexibility of CaTS and how amendments and additions can easily be made.

#### Strengths and Limitations

The primary strength of this study was the use of in-depth interviews that allowed the authors to explore factors for self-harm missing from CaTS, and amendments necessary to increase the relevance and meaningfulness of CaTS to ensure it remains a useful research tool. We were able to elicit in-depth motivations and experiences, which impact self-harm and may not have been relevant when CaTS was originally developed. Furthermore, we were able to elicit LGBTQIA+ experiences which will increase CaTS’ inclusivity and relevance to LGBTQIA+ self-harm. Qualitative research aims to create descriptions and interpretations.

Our primary limitation pertains to our sample. Significant effort was placed into recruiting transgender and gender-diverse participants. Sadly, these focused recruitment drives were largely unsuccessful. It is possible transgender and gender-diverse people experience factors for self-harm which differ from the wider LGBTQIA+ community and were not identified by participants in this study. Future research should capture transgender and gender-diverse views of CaTS and other self-harm tools to ensure they contain meaningful items which capture self-harm experiences of transgender and gender-diverse people. In addition, participants' ages ranged from 21 to 29 years. Younger adolescents, or older adults, may identify other self-harm factors or highlight amendments to CaTS, which increase its relevance and appropriateness to a wider age range. Indeed, previous CaTS research highlights differences between young (18‐25 y) and older (26‐57 y) adults' self-harm antecedents [26]. Similarly, most participants were White British. People from other racial backgrounds may experience factors specific to their racial or ethnic background that do not appear in the existing cards and task. Future research should focus on racial and ethnic groups to address this deficit. Furthermore, while some participants may have past or current clinical intervention or treatment, this sample was community-based. A clinical sample may identify additional factors relevant to clinical or in-patient self-harm experiences.

## Conclusion

These 2 studies present updates and amendments to CaTS to ensure it remains a valuable self-harm research tool. First, findings from the pilot study found the newly developed web-app version, CaTS-D, is a feasible research tool. A small decrease in mood was observed poststudy; however, given the small sample size, the study was not powered to determine whether this change is statistically or clinically significant. Overall, participants’ emotional state remained largely stable, suggesting CaTS-D can be used without substantial negative impact. Developing a feasible web-app version allows researchers to capture large datasets quickly and economically. Furthermore, it is likely preferable to participants used to interacting online, and who may find in-person research time-consuming and geographically limiting. We also identified important amendments and additions to CaTS using qualitative interviews that ensure CaTS captures relevant factors for self-harm. These include wording amendments and the addition of 13 cards, which reflect changes in our understanding of self-harm and changing experiences of people who self-harm. These evidence-based changes reflect emerging factors for self-harm that were not relevant when CaTS was originally developed and are important additions to ensure CaTS can meaningfully capture and examine self-harm antecedents in research. Future research should seek to identify whether additions or amendments specific to transgender, nonbinary, and different racial and ethnic groups could be added. In addition, adolescents and older people (>30 y) may elicit factors specific to their age group.

Finally, although VAS scores decreased slightly post-study, statistically, participants’ emotional state was not negatively affected by completing CaTS-D. However, our sample size may be too small to determine whether this reduction was statistically meaningful. A slight mood reduction may be due to the effects of thinking about self-harm experiences. Indeed, previous CaTS research noted mixed results with VAS reductions in mood shown for younger people but not for adults [26]. However, there is no evidence that partaking in self-harm research has significant negative impact on mood [59]. This is reassuring, but further studies with larger samples can confirm whether our sample was too small (low power) to detect an effect. Importantly, VAS data indicated that participants’ mood remained in the mid-range postparticipation, suggesting that CaTS-D participation did not result in substantial emotion reduction.

## Supplementary material

10.2196/71296 Multimedia Appendix 1 Original list of Card Sort Task for Self-Harm (CaTS) cards (original in-person task), questions in the Systems Usability Scale (SUS), additional quotes, well-being plan for the interview study, and the final themes identified by thematic analysis.

## Abbreviations

CaTS: Card Sort Task for Self-Harm
CaTS-D: Card Sort Task for Self-Harm—Digital
LGBTQIA+: Lesbian, gay, bisexual, transgender, queer, intersex, asexual, and other minoritized gender and sexual identities
SUS: Systems Usability Scale
VAS: visual analog scale
