# Supplementary material for: Co-Developing Content Updates for the Card Sort Task for Self-Harm–Digital (CaTS-D) With People With Lived Self-Harm Experiences: Pilot Study and Thematic Analysis
Source: JMIR Ment Health. 2025 Oct 24;12:e71296. doi: 10.2196/71296 (PMC12551937; doi:10.2196/71296)
Supplement: Multimedia Appendix 1 [file mental-v12-e71296-s001.docx]

**Multimedia 1: Supplementary materials**

**Inventory:**

**Sections**

S1: Original list of CaTS cards (original in-person task)

S2: Questions in the Systems Usability Scale (SUS)

S3: Additional quotes (Terminology updates)

S4: Additional quotes (Terminology updates regarding the terminology ‘boyfriend/girlfriend’)

S5: Additional quotes (Capturing neurodiversity data)

S6. Additional quotes (‘Before 6-months' timepoint)

S7: Additional quotes ‘I did not feel comfortable in my body’

S8: Additional quotes ‘I felt I did not fit in’

S9: Additional quotes ‘I found support/a community on social media’ and ‘Social media/online content was negatively impacting my mental health’

S10: Additional quotes ‘I had an upsetting sexual experience’ and ‘I was sexually abused or assaulted’

S11. Additional quotes ***‘***I was bullied because of my sexuality or gender identity’ and ‘I was discriminated against because of my sexuality or gender identity’

S12: Additional quotes ‘I was questioning my sexuality or gender identity’

S13. Wellbeing Plan for interview study

S14. Final themes identified by thematic analysis

**Supplementary materials:**

S1. List of CaTS cards (original in-person task)

| **Behaviors** | I am insensitive to pain; I did it on impulse without planning; I did other things to hurt myself (starved myself, drank too much); I got into trouble at home; I got into trouble at school/work; I got involved with a gang; I had access to means to hurt myself; I had unprotected sex; I isolated myself from others; I planned it carefully; I was drinking alcohol; I was drunk; I was having nightmares; I was high on drugs; I was not able to sleep; I was taking illegal drugs; I was very agitated and restless |
| --- | --- |
| **Feelings** | I did not know what I was feeling; I felt abandoned; I felt defeated; I felt depressed and sad; I felt disgusting; I felt energized; I felt exhausted; I felt hopeful about the future; I felt humiliated; I felt I could change for better in the future; I felt I could not escape from feelings or situations; I felt I would not be able to change myself in the future; I felt ignored; I felt like a burden on people; I felt like I did not belong; I felt numb; I felt rejected; I felt trapped; I felt very anxious; I felt very hopeless about the future; I felt worthless; I hated myself; I was angry; The mental pain was unbearable |
| **Events** | I discussed self-harm in a forum on the internet; I got into trouble with the police; I had a problem at school; I had a problem at work; I had an argument with my boyfriend/girlfriend; I had an argument with my friend; I had an argument with my parent/caregiver; I knew someone who was self-harming; I moved to a different home; I moved to a different school; I read about self-harm on the internet; I received no support from caregivers; I was a victim of a crime; I was being abused mentally; I was being physically abused; I was bullied; I was having problems with a close relationship; I was raped; I was rejected by my parents; I was taken in a residential care home; I was taken into foster care; Lots of people I knew were doing it; Someone close to me died; Someone close to me left; Someone I knew killed themselves |
| **Thoughts** | I wanted to die; I was not afraid of death; There was no-one to turn to for help; I could not trust anyone; I struggled to make decisions; I could not think of anything else to do; I wanted to kill myself; I could not solve a problem I faced; I could not tell anyone how I was feeling; No one listened to me or rook me seriously; I thought about being treated badly as a child; I trusted a caregiver |
| **Services and support** | Someone listened to me & took me seriously; I received therapy which helped; I received therapy which did not help; I talked to a caregiver which helped; I talked to a caregiver which did not help; I saw my GP which helped; I saw my GP which did not help; I phoned a helpline which helped; I phoned a helpline which did not help; I went to counselling which helped; I went to counselling which did not help; I read a self-help book – it helped; I read a self-help book – it did not help; I talked to a teacher which helped; I talked to a teacher which did not help; I talked to a social worker which helped; I talked to a social worker which did not help; I received help and support from a user-led service (i.e., Harmless); I depended on a caregiver for help and support |
| **Afterwards** | I went to hospital for overdose or self-injury; I felt worse after self-harm; Self-harm stopped me from killing myself; A&E staff were friendly and understanding; A&E staff were not friendly and understanding; I felt better after self-harm |
| **Timeline** | 6-months before; 1-month before; 1-week before; 1-day before; 1-hour before; I self-harmed; Afterwards |

S2. Questions in the Systems Usability Scale (SUS)

| **SUS Questions** |  |
| --- | --- |
|  | *Q1. Were instructions easy to follow?* |
|  | *Q2. Are the cards visually appealing?* |
|  | *Q3. Did the instructions make completing the task easy?* |
|  | *Q4. How easy did you find performing the task on your device?* |
|  | *Q5. I felt very confident using the web-app* |
|  | *Q6. I found the various functions in this web-app were well integrated* |
|  | *Q7. I found the web-app unnecessarily complex* |
|  | *Q8. I found the web-app very difficult to use* |
|  | *Q9. I needed to learn a lot of things before I could get going with this web-app* |
|  | *Q10.  I think that I would like to use this web-app frequently* |
|  | *Q11. I think that I would need the support of a technical person to be able to use this web-app* |
|  | *Q12. I thought the web-app was easy to use* |
|  | *Q13. I thought there was too much inconsistency in this web-app* |
|  | *Q14. I would imagine that most people would learn to use this web-app very quickly* |
|  | *Q15. Is the wording on the cards easy to read?* |

S3: Additional quotes (Terminology updates):

**Pandora:**

*"…there's, 'I discussed self- harm in a forum on the Internet' that's, that's on there, but I don't think that's as relevant now. I think that could be updated because I don't think I don't think people really use forums they use, they might be in like a Facebook group or something, or or Reddit channel and where they, you know, discuss these things instead. So...it's still relevant, but it I think the...format and which, which it’s discussed could be updated..."*

*"…I don't think people really use forums anymore... they're quite an outdated concept, but they exist, don't they? AND "Yeah...just be like a social media influencer, you know, instead of a Blogger, you know?"*

**Belle:**

*"...I think forums does happen, but maybe not so much. I think we're like platforms, social media platforms"*

**Jo:**

*"...I think you could just keep it broad like on like, 'support online' because I think that captures a range of different services or communities."*

**CH:**

*"Could be one like about Twitter instead of about forums? 'Cause I know there are like self-harm groups on Twitter and like groups for other like disorders and stuff."*

S4: Additional quotes (Terminology updates regarding the terminology ‘boyfriend/girlfriend’):

**Bronwyn:**

*"I think there's one set of cards that said something like ‘I had an argument with my boyfriend/ girlfriend’, which obviously if your partner is nonbinary or you identify in a different way, that could be something that maybe could be improved...just ‘partner’ works."*

**Selma:**

*"...There's just one thing I ... wrote down. I also wrote down, I think one was something about boyfriend and girlfriends...I think maybe for clarity's sake, just like partner. Yeah, like less words."*

S5: Additional quotes (Capturing neurodiversity data):

**Bronwyn:**

*"So, where I've been like ohh, this isn't completely clear for someone who might be really autistic then that could be something that's like I can't get past this card because I don't know what it means. UM …and that's not to say that the cards should change, but if there was a preamble or certain card that was like ‘I have this form of neurodiversity”, this is why I've interpreted it this way."*

*"...Yeah. I just wonder if it's maybe like a logging thing rather than a card thing of like, if you're gonna get some demographics beforehand, can they just type in or like click a button that says like, Yep, this neurodiversity…I don't know if the app would have this, but if there's anything that like, they want to explain the context in their own words a bit more. I found with like friends like me...people tend to want to say “by the way I have this sort of neurodiversity.” So they want to kind of explain why they're saying things in a certain way."*

*"...I feel like if I went through all of this and I'm looking at all the cards by the end of it, I'd be like, 'I don't know if I've got different experience'. I feel like it's probably been captured and maybe I do. It's just I won't have the capacity at that moment to be like, ohh yeah, actually this one very specific feeling was missing and that was key."*

*"Like, tell us about yourself…because I, I feel like if I went through all of this and I'm looking at all the cards by the end of it, I'd be like, 'I don't know if I've got different experience'. I feel like it's probably been captured and maybe I do. It's just I won't have the capacity at that moment to be like, ohh yeah, actually this one very specific feeling was missing and that was key."*

**NE:**

*"I would also say there's a lot of a lot more studies now about how neurodiversity is sort of linked to queerness. So, I think, yeah, angling some more things or just having drop-down boxes for some of those cards where you can specifically say, oh, it's because of sort of, I'm autistic or I have ADHD or something like that...that will help..."*

S6. Additional quotes (‘Before 6-months' timepoint)

**NE:**

*"I think that, yeah, just having like an extra one that's something to do with before six months, but not like a specific ‘a year before’ because...it might be something that occurred way earlier in childhood, but yeah...that would help if it was something ‘before six months’..."*

**Emily:**

*"Yeah...you've got, yeah, things like things like ‘I was raped’, ‘I was a victim of crime’ like that. They’re factors why people self-harm but could have been like way before six months? Yeah, and it's almost like, snowballed. So yeah, I definitely think a card or even a few cards like ‘before six months’ would make a lot of sense..."*

S7: Additional quotes ‘I did not feel comfortable in my body’

**Coral:**

*"...I think with social media, I guess you've got that whole thing around like, you know, comparing yourself to others, I guess that's quite a big thing. It's like kind of body dysmorphia. Kind of, yeah, it can definitely be a factor"*

S8: Additional quotes ‘I felt I did not fit in’

**Bronwyn:**

*"…I felt as though I couldn't care about myself because my identity wasn't accepted. I can't talk to anyone because I feel like they can't relate to who I am. I can't be my authentic self. Those sorts of prevalent experiences that we know happen within the community and everyone who's LGBTQ+ has a story about that in some capacity."*

S9: Additional quotes ‘I found support/a community on social media’ and ‘Social media/online content was negatively impacting my mental health’

**Belle:**

*‘”..I think social media and self-harm definitely needs to be looked into cause...it's the extremes of what they're posting about, like we have the good content are people talking about their journey and getting better and giving tips. But you have also the other side that like fuels people wanting to self-harm more because they're seeing it."*

S10:. Additional quotes ‘I had an upsetting sexual experience’ and ‘I was sexually abused or assaulted’

**Belle:**

*"...then, I did think there was like it's there's ‘I was raped’ as a card, but not necessarily, like ‘I was assaulted or sexually assaulted’ like, rape definitely is one of the cards that needs to be there, but I also think you know it doesn't necessarily need to lead all the way to rape. It can be other things that do you feel that way...the language there, like some people just aren't ready to, to class it or label it [as rape], and that's fine, but it is still like an experience you wanna like, understand and see coming through with the cards when people are using it."*

**Coral:**

*"...it goes on to saying 'I was raped', but I don't think there's a way that there could be an extra one about sexual abuse. Some people might experience sexual abuse without being raped, so they might not know how to include that, for example, but…Maybe just a bit more specific..."*

S11. Additional quotes ***‘***I was bullied because of my sexuality or gender identity’ and ‘I was discriminated against because of my sexuality or gender identity’

**Coral:**

*"...I guess some people can experience discrimination due to their sexuality and gender, I think that's in like maybe another event as well that could cause, like, trouble."*

S12: Additional quotes ‘I was questioning my sexuality or gender identity’

**Coral:**

*"...some people...but kind of like gender dysphoria or struggling with sexuality in general could be one, for example, that I know…I have lived experience with that, so I think maybe that, for example..."*

S13. Wellbeing Plan for interview study

**Participant Wellbeing Plan: Safeguarding and Managing Distress**

**Adapted suicide safety plan to address self-harm, suicide ideation, and suicidal behaviours: Participant views of the Novel Card Sort Task for Self-Harm (CaTS): An interview study**

**Objective:**

To ensure participants are supported appropriately by the research team during the study. This includes participants experiencing distress regards their own self-harm, or the self-harm of others and researcher concerns regards the safety of participants, particularly those who disclose they are at risk.

**Delivered by:**

PhD researcher and research study team, including a researcher experienced in researching self-harm.

**One-one interview sessions:**

The study will recruit participants to attend one interview session to discuss the novel card sort task for self-harm (CaTS). Participants will be asked to consider factors relating to self-harm which may affect participant wellbeing and cause distress. This may occur at any point in the study, including afterwards. Considering this, it is important to ensure participant safety as best we can. Sessions may be online or in-person. The delivery will impact how the research team are able to identify possible distress in participants, so it is important the research team pay attention to non-visual signs of distress. A Wellbeing Plan must be completed by all participants before the interview.

**Distress:**

All expressions of distress or other safeguarding issues should, and will, be taken seriously by the research team.

Distress can be expressed in many ways, including, but not limited to:

- Direct statements detailing distress
- Difficulty controlling emotions
- Difficulty controlling anger/temper
- Angry or hostile outbursts
- Yelling/shouting, or aggressing comments
- Mood swings
- Erratic or irritable behaviour
- Anxiety
- More withdrawn or animated than usual
- Expressions of hopelessness or worthlessness
- Crying or tearfulness
- Excessive demands or dependent behaviours
- Restlessness, including shakiness, tremors, fidgets, pacing
- Deteriorated physical appearance or hygiene
- Fatigue or exhaustion
- Discussing or experiencing changes in sleep
- Noticeable changes in weight, or stating changes in appetite
- Frequent or chronic illnesses
- Changes in body language
- Changes in communication style, including tone of voice, or increase/decrease in dialogue, and using more emotional or negative language
- Concentration difficulties
- Difficulties relaxing

**Wellbeing Guidance:**

The research team takes guidance from the safeguarding practices of partner organisations regarding Wellbeing plans for participants. Guidance to ensure participants wellbeing is detailed her:

- Ensure all participants complete a Wellbeing Plan (Appendix 1) prior to the session
- Encourage participants to make someone close to them aware they are taking part in the study, and they may need to call on that person
- If online, the address where the participant is taking part in the study from will be taken in case of medical emergency (this is included in the Wellbeing Plan, appendix 1)
- Participants’ GP and an ‘emergency contact’ details will be taken in case the research team is required to contact them (Wellbeing Plan, appendix 1)
- Follow-up with each participant after 24-hours to check in on them
- If a participant does show or state distress, the researcher will be sensitive and empathetic
- The researcher will identify if the person can call a trusted person (friend, family, or a healthcare professional) and encourage them to do so. If necessary, or requested by the participant, the researcher will call the nominated person
- The researcher will ask if the participant wishes to have a further follow-up call the following day
- If the researcher feels the person has an imminent plan, or is at immediate risk, they will ascertain the whereabouts of the participant and inform them they will call an ambulance
- During the study, researchers will periodically ask if anybody wants to take a break and remind participants the break-out room is available if needed
- Researchers will consider their phrasing of sentences during each session. For example, phrasing something more positively may reduce distress (I.e., “I know you are being asked to consider concepts around self-harm and this may be distressing. We really appreciate you taking time to discuss the relevance of these concepts”)
- Advise participants to avoid alcohol as this may make the situation and/or their feelings feel worse
- Participants will be advised to contact friends, family, or other support person if necessary
- Participants will be provided with general and trans-specific organisations that can provide further support:
  - Self-Injury Support: 0808 800 8088 [www.selfinjurysupport.org.uk](http://www.selfinjurysupport.org.uk/)
  - Harmless: [www.harmless.org.uk](http://www.harmless.org.uk/)
  - Samaritans: 116 123
  - LGBT Hero: 0300 330 0630 [www.lgbthero.org.uk](http://www.lgbthero.org.uk/)
  - NHS: dial **111** if not an emergency; dial **999** in an emergency; Visit A&E or another NHS urgent care in an emergency
  - Nottingham City Crisis Resolution and Home Treatment Team

CRHT City

Highbury Hospital

Bulwell

Nottinghamshire

NG6 9DR

Tel: 0300 300 0065

- If a participant indicates or suggests they are at risk of self-harm, researchers have a duty of care to take immediate and appropriate action. In this instance, the principal investigator will be informed immediately
- The research team will listen without judgment to what the person has to say and avoid making assumptions
- Information should be documented in the participants own words on the **Serious Adverse Events** form (Appendix 2. Held by research team). The event should be documented and sent to the PhD research supervisor, Ellen Townsend, within 24-hours
- If the participant is at immediate risk, or in need of immediate medical attention, action should be taken to ensure their safety and wellbeing. This may involve calling an ambulance to the address given as the current whereabouts of the participant.

Participant confidentiality can be broken if one of the research team believed a participant, or someone else, is at risk of self-harm or harm. Researchers may contact emergency services, the participants GP, or emergency contact to ensure participant safety. These will be collected in the Wellbeing Plan prior to each session. Participants will also be directed to the support services listed above.

As a participant, you will be asked to give your views on the existing, novel card sort task for self-harm (CaTS). You will also be asked for advice to refine CaTS from a trans* perspective. This will ensure CaTS is appropriate and meaningful to successfully capture the self-harm pathway experienced by trans people.

The study will involve you looking at CaTS and known factors for self-harm so you will think or hear about potentially difficult or distressing topics. Participant safety and wellbeing is paramount to our research team. We want to remind participants to prioritise their safety and wellbeing. This involves participants only contributing in ways they feel comfortable with, and that will not negatively impact their safety and wellbeing. In line with similar studies, all participants will complete a Wellbeing Plan. The Wellbeing Plan will help us safely manage any distressing situations should they arise.

Participants will be asked to complete the Wellbeing Plan below prior to the interview session. The Plan should be completed at a time you can think clearly about what you will find helpful. A copy of the Wellbeing Plan will be stored on password protected computer. Participants can share a copy of the Wellbeing Plan with a trusted person if they wish.

Your name and contact details will be kept separately from research data. 
 
Your name: 
 
Contact name: 
 
e.Mail address:

Please provide a contact number which can be used to provide a follow-up call 24-hours after the study. This is to ensure your wellbeing: 
 

The sessions will take place online (via Zoom).  
 
Do you have access to a computer or device and reliable internet to allow you to take part? 
 
The results from this interview study may be published in academic journals and conference proceedings. It would be helpful to understand a little more about you, so that a summary of group participants can be gathered. You will not be personally identified. 
 
Your age:

Your gender identity: cis female / cis male / trans female / trans male / non-binary / gender fluid / other (please specify)

Your sexuality:

Your ethnicity:

Your pseudonym: (This may be used in the research paper for anonymity purposes)

How would we, the research team, know you are becoming distressed or finding the interview difficult or challenging? 
 
What key signs should we be aware of? Some people may become quiet or agitated and feel they need to leave the interview for a while.

___________________________________________________________

How can we best support you if you do begin to feel distressed, or begin to find the interview challenging? 
 
What would you like us to do? For example, if you would like to head to a manned break-out room how you would like to let us know you need to take a break? Or, if you would like to leave the interview would you like a member of the research team to get in touch soon as possible to check in with you?

____________________________________________________________

Are there any adjustment we could make so you find participating in the interview easier for you? 
For example, would you like to know ahead of time key questions we will ask during the sessions? Or, would you like the option of writing your responses?

____________________________________________________________

What do you plan to do after the interview study? It can be helpful to plan to do something you enjoy doing after taking part in research sessions. This could be on your own or with a trusted person.

____________________________________________________________

**Sharing your wellbeing plan** 
 
**Would you like us to share a copy of your wellbeing plan with a trusted person? Yes/No** 
**(Please confirm with the person beforehand that we can contact them in order to share this information)**

**If yes, please tell us:** 
 
 
**Their name:** 
 
 
**Their relationship to you:** 
 
 
**Their phone number:** 
 
 
**Their email address:**

**In case of emergency, we need the address from where you will be taking part in the study, if online:**

**Address:**

**Postcode:**

S14. Final themes identified by thematic analysis

| **Additional cards** |  |
| --- | --- |
|  | ‘Before 6-months' |
|  | ‘I did not feel comfortable in my body’ |
|  | ‘I had an upsetting sexual experience’ |
|  | ‘I was sexually abused/assaulted’ |
|  | ‘I felt I did not fit in’ |
|  | ‘I found support/a community online’ |
|  | ‘Social media/online content was negatively impacting my mental health’ |
|  | ‘I was bullied on social media’ |
|  | ‘I was discriminated against on social media’ |
|  | ‘I was bullied because of my sexuality or gender identity’ |
|  | ‘I was discriminated against because of my sexuality or gender identity’ |
|  | ‘I was questioning my sexuality or gender identity’ |
|  | ‘Self-harm gave me a feeling of control’ |
|  | ‘Social media/online content was negatively impacting my mental health’ |
| **Amendments** |  |
| **Terminology updates** | Change ‘forum’ to ‘social media/online’ |
|  | Change ‘boyfriend/girlfriend’ to ‘partner’ |
| **Capture additional data** |  |
|  | Capture neurodiversity data |
|  | Highlight sexual violence trigger warnings’ |
